# Hysteria

**Published:** 1844-11-16

**Authors:** 


HYSTERIA.

Dr. Allnatt narrates a case of severe hysteria with ptosis, caused by a flash of lightning, which he cured by purgatives, tonics, and sea-bathing. The complaint was removed in about a month, or six weeks. Dr. Allnatt details the case to urge the necessity of caution in forming a diagnosis, as much injury might have accrued from an erroneous opinion in this case. Cases of hysteria mistaken for inflammatory action are by no means rare.—*Ibid.*

